# Three-dimensional printed custom-made modular talus prosthesis in patients with talus malignant tumor resection

**DOI:** 10.1186/s13018-024-04728-6

**Published:** 2024-05-02

**Authors:** Xuanhong He, Minxun Lu, Chang Zou, Zhuangzhuang Li, Taojun Gong, Guy Romeo Kenmegne, Yitian Wang, Yi Luo, Yong Zhou, Li Min, Chongqi Tu

**Affiliations:** 1 Department of Orthopedics, Orthopedic Research Institute, Trauma Center West China Hospital, Sichuan University, No. 37 Guo Xue Xiang, Chengdu, 610041 Sichuan People’s Republic of China; 2 Department of Model Worker and Innovative Craftsman, West China Hospital, Sichuan University, No. 37 Guoxuexiang, Chengdu, 610041 Sichuan People’s Republic of China

**Keywords:** Three-dimensional printed, Custom-made modular talus prosthesis, Talus tumor, Reconstruction

## Abstract

**Background:**

Talar malignant tumor is extremely rare. Currently, there are several alternative management options for talus malignant tumor including below-knee amputation, tibio-calcaneal arthrodesis, and homogenous bone transplant while their shortcomings limited the clinical application. Three-dimensional (3D) printed total talus prosthesis in talus lesion was reported as a useful method to reconstruct talus, however, most researches are case reports and its clinical effect remains unclear. Therefore, the current study was to explore the application of 3D printed custom-made modular prosthesis in talus malignant tumor.

**Methods:**

We retrospectively analyzed the patients who received the 3D printed custom-made modular prosthesis treatment due to talus malignant tumor in our hospital from February 2016 to December 2021. The patient's clinical data such as oncology outcome, operation time, and volume of blood loss were recorded. The limb function was evaluated with the Musculoskeletal Tumor Society 93 (MSTS-93) score, The American Orthopedic Foot and Ankle Society (AOFAS) score; the ankle joint ranges of motion as well as the leg length discrepancy were evaluated. Plain radiography and Tomosynthesis-Shimadzu Metal Artefact Reduction Technology (T-SMART) were used to evaluate the position of prosthesis and the osseointegration. Postoperative complications were recorded.

**Results:**

The average patients’ age and the follow-up period were respectively 31.5 ± 13.1 years; and 54.8 months (range 26–72). The medium operation time was 2.4 ± 0.5 h; the intraoperative blood loss was 131.7 ± 121.4 ml. The mean MSTS-93 and AOFAS score was 26.8 and 88.5 respectively. The average plantar flexion, dorsiflexion, varus, and valgus were 32.5, 9.2, 10.8, and 5.8 degree respectively. One patient had delayed postoperative wound healing. There was no leg length discrepancy observed in any patient and good osseointegration was observed on the interface between the bone and talus prosthesis in all subjects.

**Conclusion:**

The modular structure of the prosthesis developed in this study seems to be convenient for prosthesis implantation and screws distribution. And the combination of solid and porous structure improves the initial stability and promotes bone integration. Therefore, 3D printed custom-made modular talus prosthesis could be an alternative option for talus reconstruction in talus malignant tumor patients.

## Introduction

Foot is an extremely rare site of occurrence for malignant tumors [1–3]. The most common malignant tumors invading foot are chondrosarcoma, Ewing's sarcoma and osteosarcoma [4]. Among foot malignant tumors, talus malignant tumors occupy a smaller part of 0–15% [1, 3–6]. The below-knee amputation or pirogoff amputation are the common treatment options for hindfoot malignant tumors; however, limb loss and reduced quality of life are the main concerns of those management approaches [7, 8]. With the advancement of systemic treatment and surgical techniques, the possibility of tumor wide resection and limb preservation has greatly increased [8–10]. Unfortunately, limb preservation equally revealed new clinical dilemma. Tibiocalcaneal fusion has to sacrifice the ankle joint function with risk of limb shortening [11–13]. Frozen autologous bone graft reconstruction approach is challenged by, low bone strength, delayed bone healing, and the complications related to prolonged postoperative limb immobilization [14–16].

With the development of digital orthopedics and additive manufacturing technologies, three-dimensional printed (3D printed) talus body prosthesis or total talus prosthesis has been gradually applied in talus reconstruction [17–19]. In patients managed with 3D printed talus prosthesis following talus necrosis, it was reported a certain extent of joint mobility recovery; significant improvement of the limb function with early weight-bearing activities allowed and negligible leg length dis crepancy [20–24]. However, complications such as distal tibia sclerosis, prosthesis loosening or displacement, and infection were reported during follow-up [25, 26]. Moreover, due to the rarity of talus malignant tumors, there are very few reports on the reconstruction of the talus [7]. Additionally, most literatures are confined to the case reports, resulting in the unclear clinical efficacy of 3D printed talus prosthesis approach in management of talus malignant tumor patients [17–19, 27].

In this study, we developed a novel 3D printed custom-made modular talus prosthesis for patients with talus malignant tumors. Based on the CT data of the contralateral talus, the prothesis was well matching with the distal tibia and adjacent foot bones. The prosthesis was composed of an ultra-high molecular weight polyethylene (UHMWPE) part and titanium alloy part (Ti6Al4V powder, Chunlizhengda Corp., Beijing, China). The polyethylene part can reduce the wear on the tibia side, while the titanium alloy part has a solid inner layer and a porous outer layer which ensures the immediate stability, improving the bone integration and enhancing the long-term stability of the prosthesis. In order to further determine the clinical efficacy of this 3D printed custom-made modular talus prosthesis in talus malignant tumors, this study retrospectively recorded the patients’ data, assessed their oncological outcome, limb function, as well as the potential associated postoperative complications.

## Methods

### Patients

Between February 2016 and December 2021, six patients with talus malignant tumor underwent enlarged talus tumor resection and reconstruction with 3D printed custom-made modular talus prosthesis. The inclusion criteria included: (1) talus malignant tumor; (2) good skin and soft tissue conditions; and (3) the willing to bear the potential risk of three-dimensional printed custom-made modular talus prosthesis. The exclusion criteria for participating in this study were as follows: (1) Tumors braking through the compartments and invading adjacent bones; (2) recurrent or metastasized tumor; (3) patient’s poor medical condition and patients who could not tolerate the surgical procedure. Patients with such conditions needed amputation: tumors invading extensive adjacent bones and tissue or with multiple recurrences which could not acquire a margin-nagative resection needed amputation; tumor eroded the surrounding vital nerve and vessels; tumors were insensitive to chemotherapy or other system management methods and had a trend of progression. Finally, there were two males and four females eligible for the study enrolled, and retrospectively evaluated.

All patients received en bloc resection and reconstruction with three-dimensional printed custom-made total talus prosthesis. After surgery, all patients underwent plain radiography (PR) and Shimadzu Metal Artefact Reduction Technology (T-SMART) of the ankle monthly in the first 3 months and trimonthly afterwards. The Musculoskeletal Tumor Society 93 (MSTS-93) and The American Orthopedic Foot and Ankle Society (AOFAS) criteria were used to evaluate the limb function. The ankle-joint range of motion was recorded. Among six patients, two patients were initially diagnosed as chondrosarcoma, while the four others had distinct diagnoses including mesenchymal sarcoma, Ewing sarcoma, malignant spindle cell proliferative tumor, and osteosarcoma, respectively. The VDC/IE chemotherapy regimen included vincristine, doxorubicin, cyclophosphamide, ifosfamide and etoposide; The AP chemotherapy regimen included doxorubicin and cisplatin. The patient with Ewing sarcoma received in total 12 cycles of the VAC/IE chemotherapy pre and postoperatively. The patient with osteosarcoma received AP chemotherapy for a total of 12 cycles during the management process. The median follow-up duration was 54.8 months. None of the six patients presented the evidence of disease reoccurrence or dysfunction during and at the last follow-up evaluation.

This study was approved by the Ethical Committee of West China Hospital. All patients agreed to participate in this study and signed the written informed consent.

### Design and fabrication of the total talus prosthesis

**Fig. 1:**
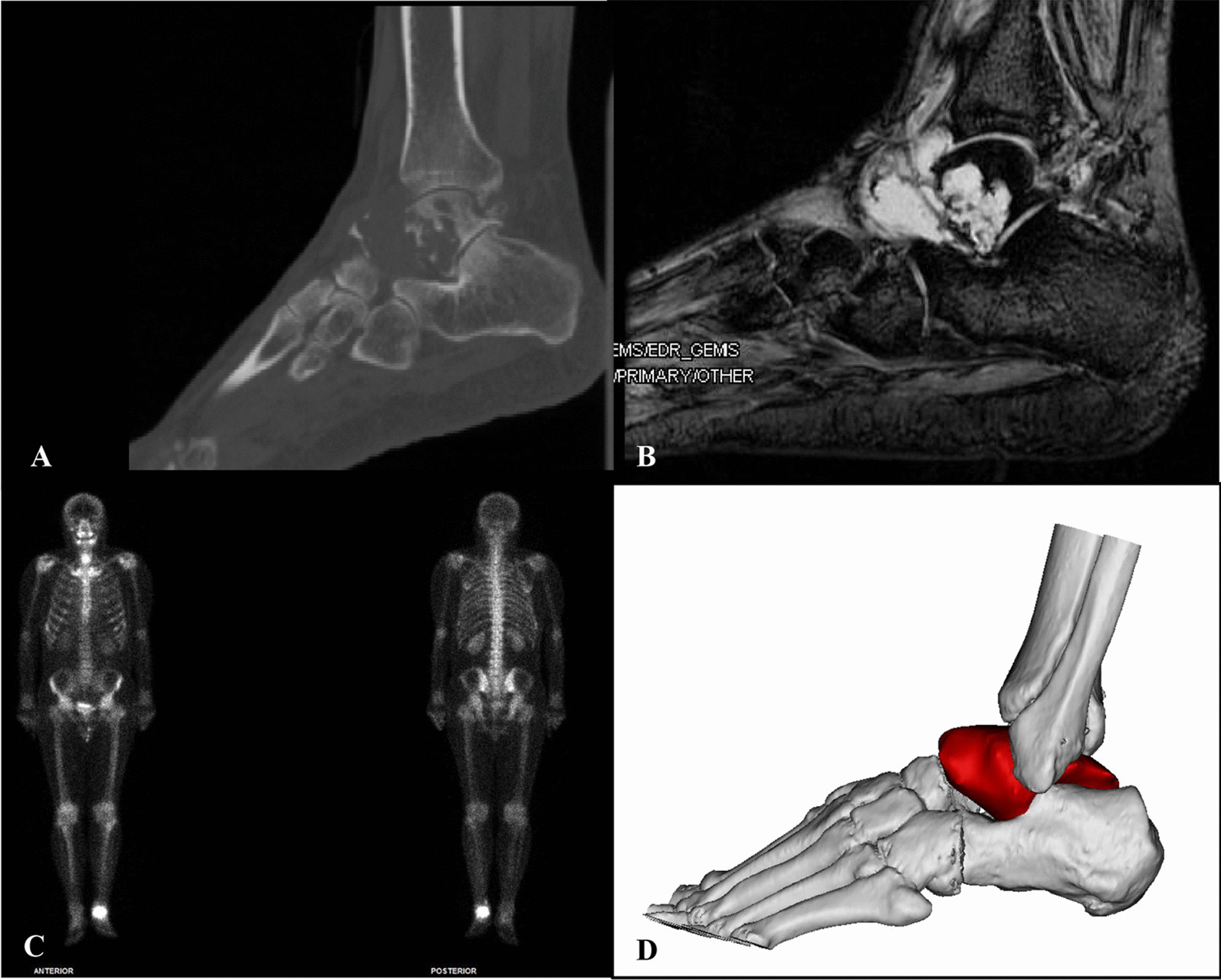
Preoperative imaging including CT (**A**), MRI (**B**), and SPECT (**C**) tumor model (**D**) of the patients

Plain radiography, CT, MRI and SPECT scan were performed to evaluate the preoperative tumor edge (Fig. 1). The CT data of patients were imported to Mimics V20.0 software (Materialise Corp., Leuven, Belgium) to simulate virtual three-dimensional models of the tumor and bone. The resected tumors and the residual bones were rebuilt and the operation procedure were simulated in the Geomagic Wrap software (Geomagic inc., Morrisville, NC) (Fig. 1).

**Fig. 2:**
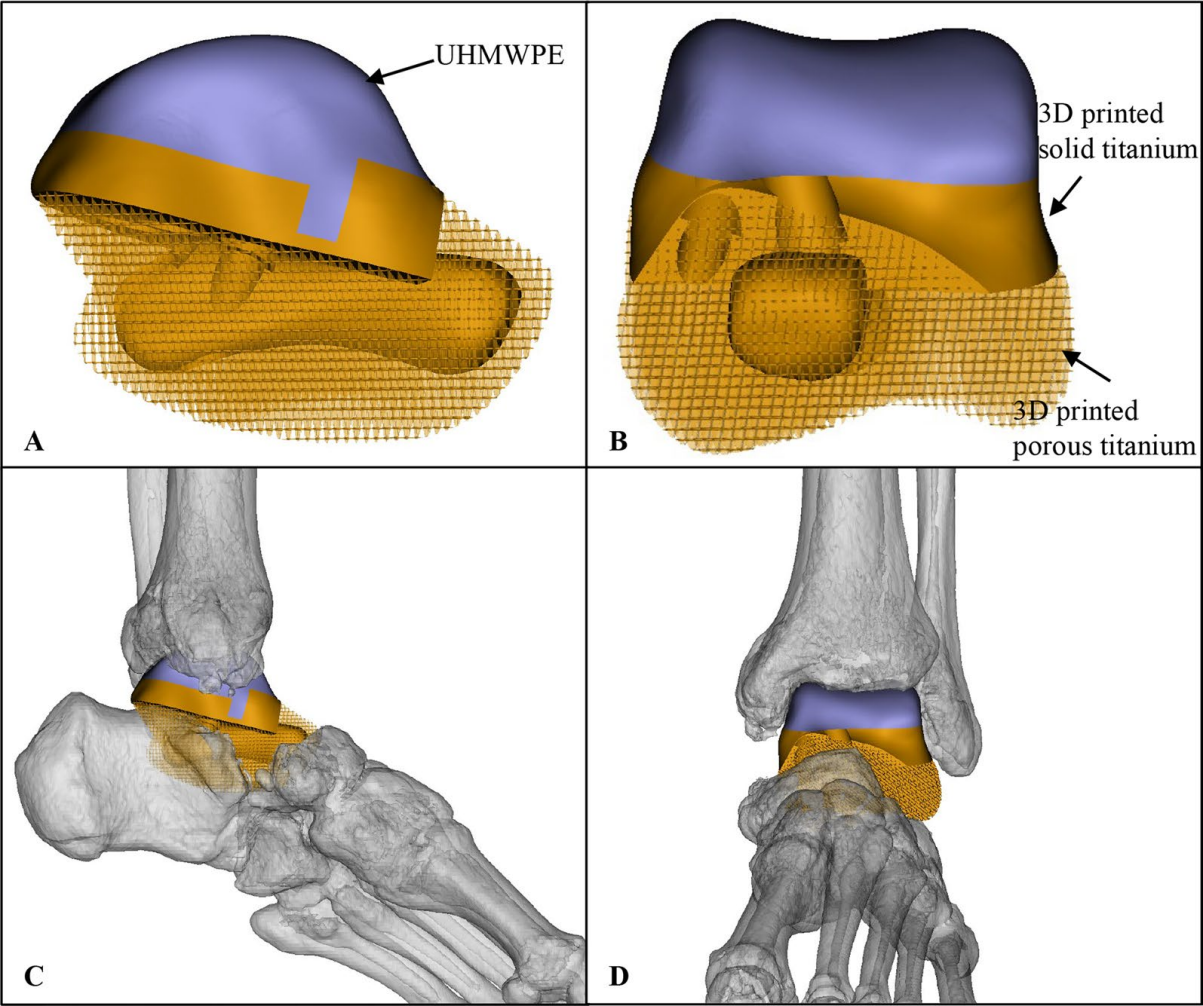
Design configuration of the 3D-printed custom-made modular talus prosthesis. **A**, **B** The prosthesis is composed of an ultra-high molecular weight polyethylene (UHMWPE) parts and titanium alloy part. **C**, **D** Simulation of the prosthesis implantation following tumor resection

**Fig. 3:**
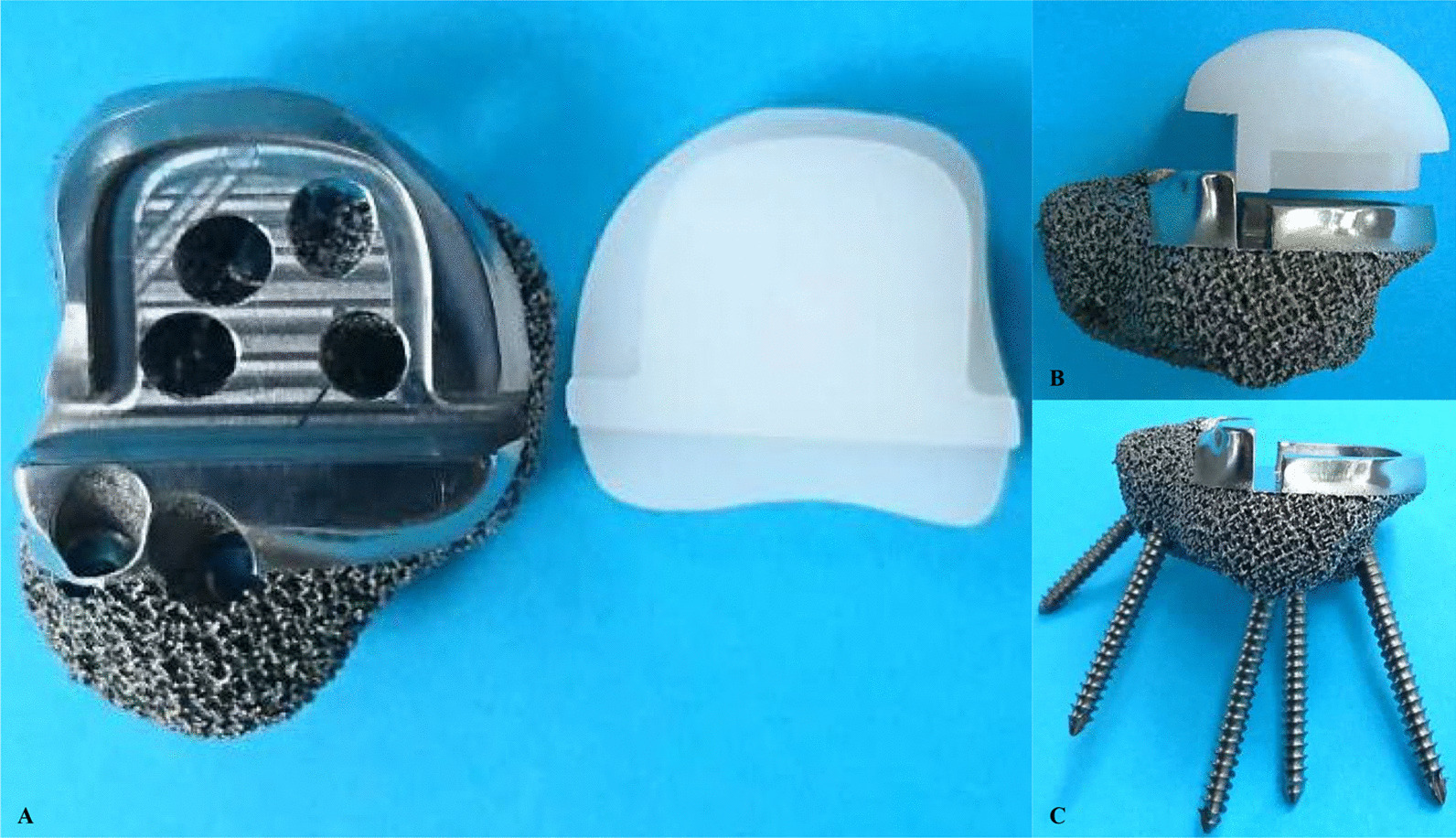
The fabrication of the 3D-printed custom-made modular talus prosthesis. **A**, **B** Screw holes are pre-set in the titanium alloy parts for screw insertion. The UHMWPE part and the titanium alloy parts were connected according to the “snap-fit” design. **C** The screws are evenly distributed

All the three-dimensional custom-made modular talus prostheses were designed by our institution’s clinical team on the basis of the healthy side talus anatomical data, and were manufactured by Chunli Co., Ltd., Tongzhou, Beijing, China. The shape of the talus prosthesis was created by mirroring the shape of the healthy talus. The modular prosthesis consisted of a UHMWPE and a titanium alloy parts, connected by a "snap-fit" design. The titanium alloy part has a solid titanium structure and porous titanium structure respectively in the inner and the outer layer, with a pore size of 600 μm with an average porosity of 50–80%. The titanium alloy part was designed with screw holes for fixation of the prosthesis to the surrounding bony structures (Figs. 2, 3).

### Surgical procedure

**Fig. 4:**
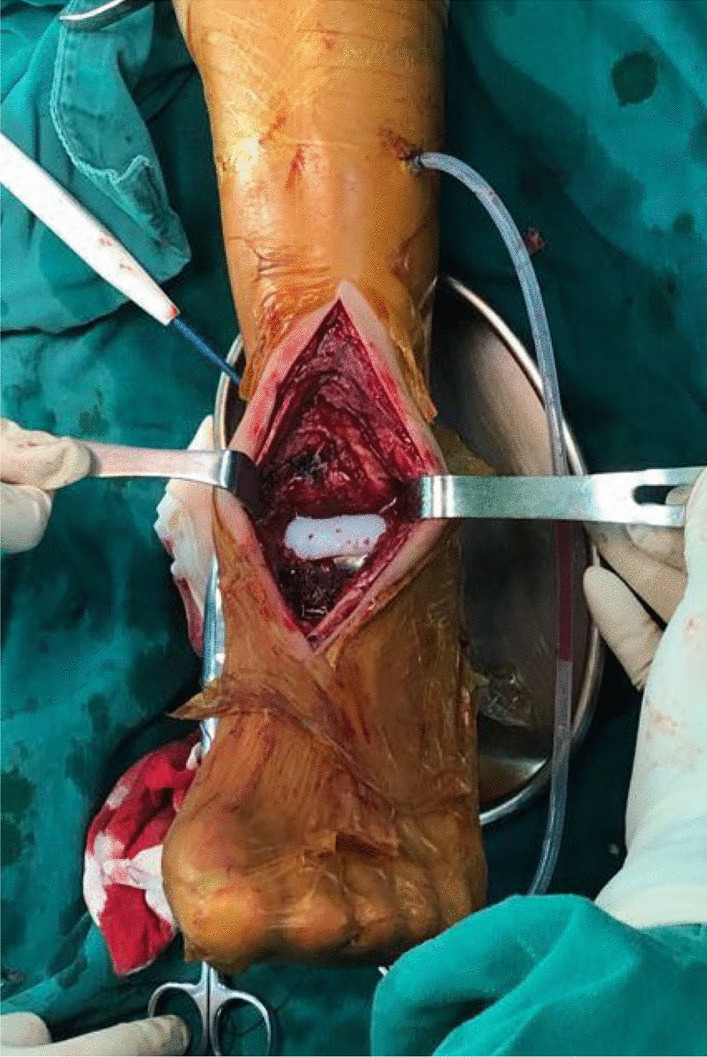
Intraoperative tumor resection and implantation of the 3D-printed custom-made modular talus prosthesis

Patients were placed in supine position and a tourniquet was applied on the affected side thigh. The talus was exposed through the anterior approach between the tendons of the extensor digitorum longus and fibularis longus. The soft tissues were stripped after separating and protecting the medial branch of the superficial peroneal nerve and the anterior tibial artery. According to the Enneking staging, all the patients were underwent extensive or radical resection. Following complete resection of the talus bone, the operative area was alternately soaked and irrigated with 10% povidone-iodine solution; the surrounding tissues were handled with electrosurgical cauterization. The subtalar cartilage of the calcaneus was removed for facilitating arthrodesis. The metal modular components of the prosthesis were installed to the calcaneus and navicular bones with respect to the prior preoperative simulations. Screws were placed in three directions to secure the subtalar joint through pre-set holes. The UHMWPE component was finally pressed in and fitted to the metal module component, following with squirtile irrigation, wound soaking/irrigation using 10% povidone-iodine solution for 3 min, and another squirtile lavage. A drainage tube was placed and pressure bandage was applied after wound closure (Fig. 4).

Postoperatively, the ankle joint was immobilized with plaster cast; the foot required to be maintained in dorsiflexion. Range of motion exercise began at postoperative week two while weight bearing was gradually increased from 4 weeks after surgery.

### Statistical analysis

IBM SPSS Statistics software, version 22 (IBM SPSS, Armonk, NY, United States) were used to perform Statistical analyses. Continuous data are represented as mean ± standard deviation. Student’s t-test was used to compare continuous variables. *p* < 0.05 was considered statistically significant.

## Results

**Fig. 5:**
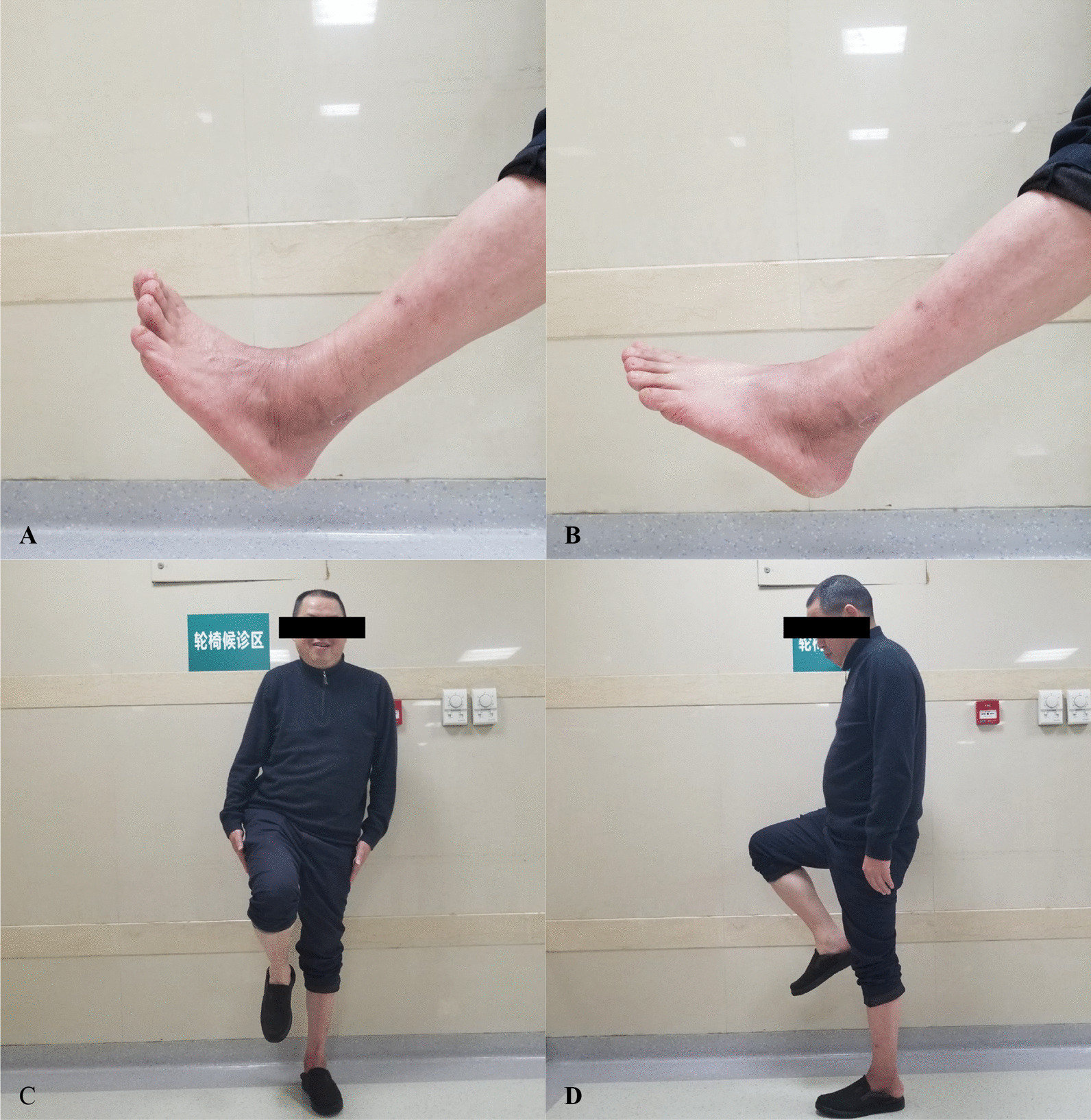
Limb function and ankle range of motion. **A**, **B** Dorsiflexion and plantar flexion of the ankle. **C**, **D** demonstrate the patient on full weight-bearing on the affected limb

**Fig. 6:**
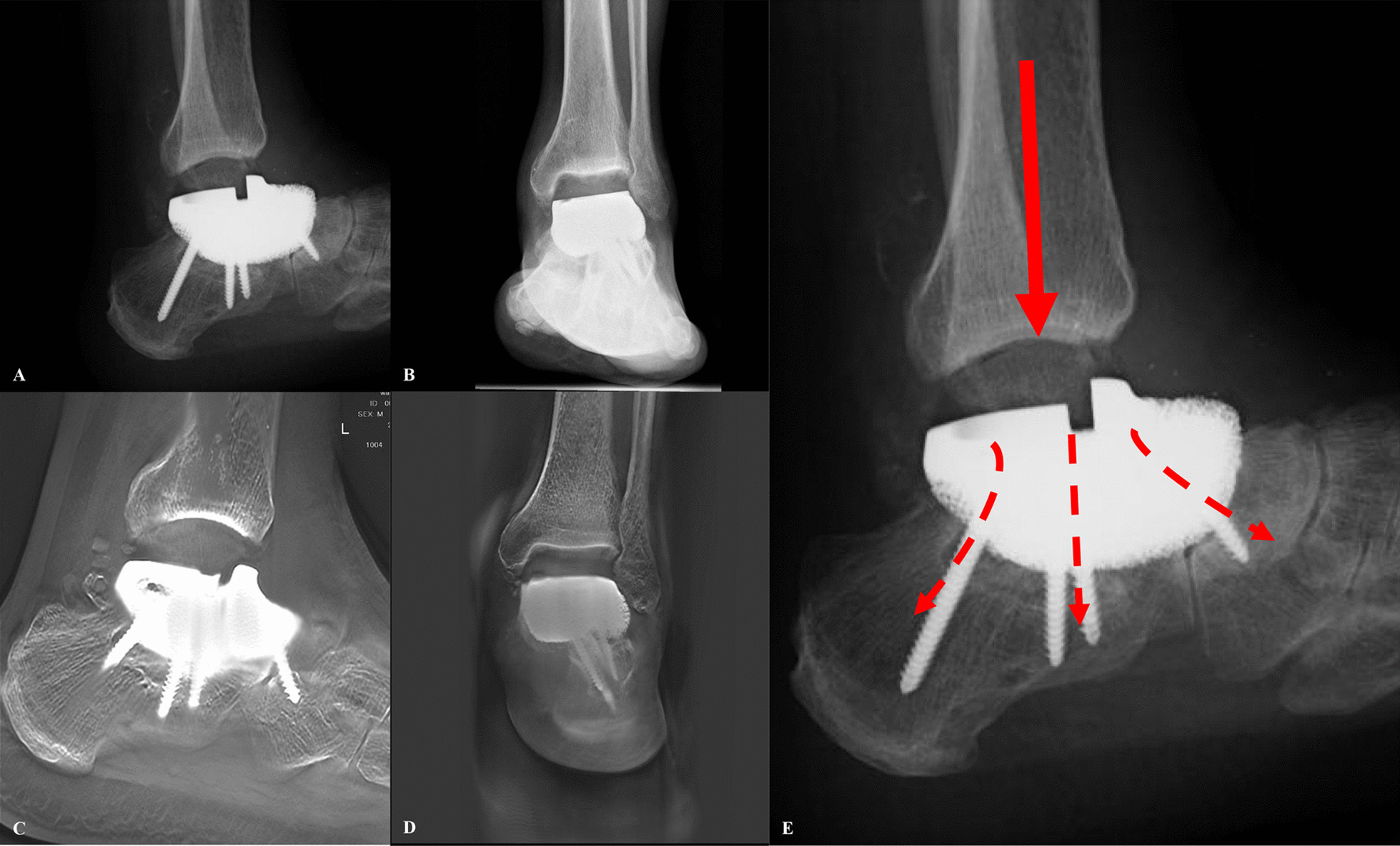
Postoperative radiographs. **A**, **B** The X-rays demonstrates the proper position of the 3D-printed custom-made modular talus prosthesis; **C**, **D** T-SMART showed the good integration between bone and prothesis. **E** Schematic diagram of weight transmission and distribution

The average age of the six patients was 31.5 ± 13.1 years; the mean follow-up time was 54.8 months (range 26–72). The operation duration was 2.4 ± 0.5 h with an average volume of blood loss of 131.7 ± 121.4 ml. The mean MSTS-93 and AOFAS score was 26.8 and 88.5, respectively. The average plantar flexion, dorsiflexion, dorsiflexion, and varus of ankle were respectively 32.5, 9.2, 10.8, and 5.8 degree (Fig. 5, Table 1). At the last follow-up, no aseptic loosening, fracture/dislocation of the prosthesis or screws loosening was observed. Delayed wound healing after operation occurred in one patient; however, it was recovered after continuous dressing changing. There was no leg length discrepancy observed; neither local recurrence nor distant metastasis was found. The T-SMART on final follow-up demonstrated that all prostheses presented good osseointegration. Plain digital radiography images revealed normal positioning without displacement or loosening of the prostheses in all the study participants (Fig. 6).

**Table 1:** Demographics of the 6 patients underwent talar resection and 3D-printed custom-made modular talus prosthesis reconstruction

No.	Age (years)	Gender	Diagnosis	Operation time (h)	Blood loss (ml)	Follow-up (month)	Oncologic outcome	MSTS-93	AOFAS	Ankle motion (degree)			
										Dorsiflexion	Plantar flexion	Eversion	Inversion
1	43	F	Chondrosarcoma	2	50	72	NED	26	91	30	10	5	10
2	14	F	Ewing sarcoma	2	100	56	NED	25	87	30	5	5	10
3	31	M	Chondrosarcoma	3.5	400	41	NED	27	90	40	15	10	15
4	51	M	Malignant spindle cell proliferative tumor	2.1	100	71	NED	28	90	35	5	5	10
5	17	F	Osteosarcoma	2.4	80	63	NED	27	86	30	10	5	10
6	33	F	Chondrosarcoma	2.3	60	26	NED	28	87	30	10	5	10
Median	31.5			2.4	131.7	54.8		26.8	88.5	32.5	9.2	5.8	10.8

F, female; M, male; NED, no evidence of disease; MSTS, Musculoskeletal Tumor Society; AOFAS, American Orthopedic Foot and Ankle Society Score

## Discussion

Foot is the rare site of malignancy and accounts for about 5 percent of malignant bone tumors [28, 29]. Data reporting the incidence of foot malignant tumors varied in literatures, with chondrosarcoma being the most reported tumor, followed by Ewing's sarcoma and osteosarcoma [1, 3, 4]. Talus malignant tumors account for 0–15% of all malignant tumors of the foot, which are considered rarer compared with calcaneus, tarsal, and metatarsal bones [1, 3–6]. Giving the feature of the foot compartments that allows rapid spread of malignant lesions, aggressive surgical treatment is necessary in talus malignancy to prevent recurrence or distal metastasis [4].

In recent years, advances in comprehensive treatment and operation techniques have made limb-sparing surgery possible [7, 8, 30]. Tibiocalcaneal arthrodesis and frozen autologous bone graft are common reconstruction approaches after talus resection. Nevertheless, the disadvantages such as loss of ankle function, limb shortening, failure of bone healing, and the prolonged immobilization have limited the clinical application [31–33]. To address those shortcomings, talus prosthesis are being designed in respect to their advantages such as joint motion preservation, early weight-bearing, rapid pain relief and preservation of limb length [26]. Our center previously reported 3D printed talus prosthesis in the treatment of talus malignancy [27]. The crucial point of the talus prosthesis application depends on the prosthesis design, the deltoid ligaments tension, and the appropriate patient selection [25]. For talus malignancy, the absences of extra-talar bone invasion or metastasis and effective chemotherapy/radiotherapy are prerequisites for the selection of 3D printed talus prostheses. Preoperative comprehensive assessment of the tumor edge based on imaging should be performed, along with a detailed surgical planning, including the surgical approach and the simulated prosthesis implantation [34, 35]. In our center, at least three experienced orthopedic oncology surgeons are required to comprehensively evaluate the resection area and the surgical approach preoperatively. Meanwhile, in the current study, additional 3D imaging techniques was applied to simulate the prosthesis implantation (Fig. 2).

Due to the special anatomy of talus, the reconstruction of talus is essential for the ankle joint stability recovery and the limb functional restoration [36]. Nonetheless, application of talus prosthesis may face several problems. For example, a mismatched prothesis often requires a larger incision or extreme plantar flexion for implantation, which may lead to extensive soft tissues injury and prolonged operation time. In addition, the stability of most total talus prostheses depends on the fixation with surrounding bones, residual ligaments and capsule, which are achieved through screw or additional stem fixation [8, 25, 26]. During the long-term follow up, there are serious concerns with foot pain due to the displacement and loosening of the prosthesis [8, 25]. In the study conducted by Harnroongroj [25], 33 patients with talus necrosis were treated using talus prosthesis; the front of the prosthesis was fixed by the talus stem and the bone cement infiltrated within the specific fixation hole in the retained talus head; the authors reported an average AOFAS score as > 76. However, during the follow-up, the talus joint was observed displacing forward, leading to the prosthesis failure and forefoot pain [25]. Moreover, this prosthesis was not suitable for most patients with talus malignant tumor as there is not enough residual bone for the talus stem to be adequately inserted and fixed [25]. Papagelopoulos et al. designed a talus prosthesis to treat talus Ewing's sarcoma, with a stem facing calcaneus for prosthesis fixation and a canal was intra-operatively created on the calcaneus to provide room for stem fixation [8]. Yet, this application of this prothesis may cause the bone loss or defect. In addition, other total talus prostheses with pre-designed subtalar or talo-navicular screw holes to improve the fixation and support of the prosthesis [19, 37]. Nevertheless, the distribution of screws is unevenly distribution with inadequate of pressure gravity sharing; the prosthesis may have poor fixation effect and conduce to screw failure under the condition of long-term uneven stress distribution [19, 37]. In particular, for talus malignant tumors, the soft tissues resection is more extensive and the requirement of prosthesis stability becomes higher, in which the fixation with surrounding bone and few soft tissues might be ineffective. Additionally, most prostheses are metal structures; the wear of metal prostheses and adjacent bones leads to osteolysis and degeneration of adjacent joints [8, 20–26, 37, 38]. Meanwhile, the metal prosthesis might not integrate with the surrounding bone and may lead to prosthesis subsidence or peri-prosthetic fracture during follow-up; these are considered as important challenges to the prosthesis life and its stability [17, 22, 23, 26].

On the basis of the previously designed prosthesis, we made some modification to improve the stability and survival rate of prosthesis. In the current study, the reported prosthesis is a modular talus prosthesis combined with a UHMWPE and a metal module component, connected by a "snap-fit" structure (Fig. 2). This prosthesis can be installed in modular intra-operatively to reduce the installation time, the scope of exposure, and the related soft tissue damages. In the meantime, the metal component was initially installed to provide more space for placing screws, allowing the position of screws to be allocated more reasonably. The three directions of the screw refered to the distal, middle and proximal directions of prothesis as displaced on the X-ray (Fig. 6). We thought that such a screw distribution could better disperse the transmitted gravity. At the same time, such reasonable nail placement ensured the stability of the prosthesis in different directions, and prevent the stability discrepancy of different part of the prosthesis caused by unilateral screw fixation, which might reduced the possibility of the loosening of the prosthesis (Fig. 6). At the last follow-up, no sinking, displacement, or peri-prosthetic fracture of prothesis was observed in our series (Fig. 6). What’s more, the UHMWPE component of the modular talus prosthesis is less abrasive to the tibial articulating surface compared with total talar prothesis [23]. There was no evidence of sclerosis of the distal tibia observed during the follow-up (Fig. 6). Worth noting, the modular talus prosthesis has the advantage in revision if necessary. During revision, the clinicians just need to replace the UHMWPE component with a new one instead of the entire prosthesis, causing less soft tissue trauma barely compromising the stability of the subtalar and talocaval joints. Additionally, metal component of the prosthesis consists of inner titanium alloy solid structure and outer porous structure, which provides initial stability while allowing osteointegration and improving long-term stability (Fig. 2). The T-SMART at the last follow-up demonstrated good bone integration between the bone tissue and the prosthesis with no gaps, translucent bands, or dark areas at the interface. Plain radiographs revealed normal position of the prosthesis with no obvious displacement or loosening (Fig. 6). At the final follow-up, the AOFAS and MSTS-93 scores in our patients was 26.8 and 88.5, comparable to the previously reports, indicating that this prosthesis is an effective substitute for malignant tumors of the talus [17–19, 27, 39, 40].

Infection is another issue in need of attention because deep infection may cause implant failure and can even be life-threatening [41]. Enlonged operation time, immune-compromising management, and limited soft tissue coverage were related to the infections in patients with malignancies [42, 43]. In this study, only a patient occured delayed wound healing without any deep infection and recovered after continuous dressing changing. There were some measures to reduce chances of infection. First, the design of the modular structure of the talus prosthesis helped to simplify the procedure of reconstruction and shorten the operation time (Fig. 3). Besides, the strict sterile operation and repeated irrigation with saline and 10% povidone-iodine solution during operation were also improtant. Finally, the correction of systemic condition before surgery and the application of antibiotics postoperative could decrease the risk of postoperative infection in patients with malignant tumor.

We have to admit that our study contains some shortcomings. Limited by the rarity of malignant tumors of the talus, there were fewer cases and shorter follow-up in our study. However, two patients in our series had a maximum follow-up time of more than 5 years, and none of them experienced tumor recurrence. Meanwhile, good limb function and excellent imaging outcomes indicate the feasibility of this talus prosthesis. Moreover, to the best of our knowledge, we are the largest reported series of 3D printed custom-made talus prosthesis for talus malignant tumors, conferring to this report, certain reference significance for the clinical application of the prosthesis. In the future, further enrollment and evaluation of patients with talus malignancy will be undertaken to strengthen the results of the current study, validate the clinical efficacy of our designed prosthesis and contribute to improving literature about talus malignancy.

In conclusion, this retrospective analysis of six talus malignancy patients demonstrated that our 3D printed custom-made modular talus prosthesis could restore joint stability and integrity thus improving the limb function. The modular structure facilitates prosthesis implantation, screw distribution; the combination of solid and porous structures improves initial stability and promotes osseointegration. Therefore, this prosthesis may be a valid and sound option for talus reconstruction in talus malignant tumors.
